# Subchronic olanzapine exposure leads to increased expression of myelination-related genes in rat fronto-medial cortex

**DOI:** 10.1038/s41398-017-0008-3

**Published:** 2017-11-30

**Authors:** Kari M. Ersland, Silje Skrede, Christine Stansberg, Vidar M. Steen

**Affiliations:** 1 Dr. Einar Martens Research Group for Biological Psychiatry, Center for Medical Genetics and Molecular Medicine, Haukeland University Hospital, Bergen, 5021 Norway; 2 The Norwegian Centre for Mental Disorders Research (NORMENT) and the K.G. Jebsen Centre for Psychosis Research, Department of Clinical Science, University of Bergen, Bergen, Norway

## Abstract

Schizophrenia is a psychotic disorder with severe and disabling symptoms, such as hallucinations, delusions, blunted affect and social withdrawal. The neuropathology remains elusive, but disturbances in immunity-related processes, neuronal connectivity and myelination have consistently been linked to schizophrenia. Antipsychotic drugs can be efficient in reducing symptoms, acting primarily on the dopamine system, but additional biological targets are likely to exist. Here we have screened for novel mechanisms of action in an animal model, using adult rats exposed to long-acting olanzapine, achieving stable and clinically relevant antipsychotic drug concentrations. By microarray-based examination of global gene expression in the fronto-medial cortex, at the single gene- and gene-set level, we observed downregulation of two neuropeptide-encoding genes, Vgf and Cort (fold change −1,25 and −1,48, respectively) in response to olanzapine exposure. Furthermore, we demonstrated significant upregulation of five out of ~2000 GO predefined gene sets after olanzapine exposure. Strikingly, all were linked to myelination and oligodendrocyte development; “Ensheathment of neurons”, “Axon ensheathment”, “Myelination”, “Myelin sheath” and “Oligodendrocyte development” (FDR-values < 25). Sixteen of the leading edge genes in these gene sets were analysed independently by qPCR, of which 11 genes displayed significant upregulation, including *Plp1*, *Mal*, *Mag* and *Cnp* (fold change: 1,30, 1,50, 1,30 and 1,15, respectively). Several of the upregulated genes (e.g. *MAG*, *MAL* and *CNP*) have previously been reported as downregulated in post-mortem brain samples from schizophrenia patients. Although caution needs to be taken when extrapolating results from animal studies to humans, the data suggest a role for olanzapine in alleviating myelination-related dysfunction in schizophrenia.

## Introduction

Schizophrenia is a disabling psychiatric disorder with a lifetime prevalence of about 0.7%^1^. The illness is characterised by complex clinical symptoms, including hallucinations, delusions, social withdrawal and blunted affect, in addition to cognitive impairment. The onset of schizophrenia is normally during late adolescence or early adulthood, a time period coinciding with brain maturation, including myelination. Although the clinical manifestations have been known for centuries, the underlying pathophysiology and aetiology of the disorder remain largely unknown. While the estimated heritability ranges from 60 to 80%^2,3^, and recent studies have started to disclose genetic risk factors of schizophrenia^4–8^, much still remains to be elucidated.

As an alternative approach to study the pathological disease mechanisms, large-scale gene expression studies have been performed on post-mortem brain samples from patients suffering from schizophrenia. The most consistent observations from these studies include alterations in expression levels for genes related to brain white matter and oligodendrocytes, signalling and synapses, GABA and glutamate neurotransmission, mitochondrial function, in addition to immune and stress-response (reviewed in^9–11^). In particular, several studies have reported a downregulation of genes linked to myelination and myelin-producing oligodendrocytes in schizophrenia^12–16^. Oligodendrocyte membranes wrap around axons forming a multi-lamellar myelin structure, which is essential for rapid propagation of action potentials and the long-term integrity of neuronal axons. Since myelin is a lipid-rich membrane structure, it is interesting that several antipsychotic drugs, especially clozapine and olanzapine, have been shown to have lipid-stimulating effects.

The results from post-mortem brain studies have, however, been challenging to replicate, probably attributed to cofounding factors, e.g. age, severity of disease, treatment with antipsychotic drugs, co-morbidity, post-mortem sample handling and type of microarray platform used^9^. Obstacles such as these are less prominent when using animals to explore drug-induced changes in gene expression in the brain, although such models have obvious limitations in the study of psychiatric disorders^17^. Rodents, primarily rats, have been extensively used to analyse the effect of various antipsychotic drugs on gene expression in different areas of the brain. Overall, the alterations identified seem to converge on some of the same pathways as observed in patient post-mortem studies, including neurotransmission, neural plasticity, cell survival, ionic homoeostasis and synaptic functions^11,18–21^.

Still, direct comparison between animal studies have been problematic due to differences in methodology, brain areas examined, choice of antipsychotic drugs and treatment schemes. The rapid metabolism of antipsychotic agents in rodents represents an additional challenge, impending clinically relevant serum concentrations of antipsychotic drugs, especially over time. As an example, the half-life (*t*_1/2_) of the second-generation antipsychotic olanzapine in rat serum is 3 h, while the average *t*_1/2_ is 30 h in humans^22–24^. Most global gene expression studies to date have used daily oral administration of antipsychotic agents (mainly through drinking water, but also by gavage) or daily injections. Recently, our group demonstrated that the use of depot injections of atypical antipsychotics in rat leads to clinically relevant and stable serum concentrations over time, as compared to the fluctuating and often low serum drug concentrations observed in rats exposed to twice-daily oral drug administration during the same time period^25–28^.

In this work, we exposed rats to a 5-day treatment with long-acting olanzapine to examine alterations in global gene expression in the fronto-medial parts of the cerebral cortex, identifying drug-induced molecular profiles. We chose to study olanzapine, since this antipsychotic drug has been shown to be one of the most efficacious antipsychotic agents, in addition to displaying strong lipid-stimulating effects^29,30^. We demonstrate a significant downregulation of two neuropeptide-encoding genes, and interestingly, a striking upregulation of gene sets linked to myelination-related functional pathways. To our knowledge, this is the first study to report on global gene expression alterations in rat brain in response to stable and clinically relevant olanzapine levels.

## Materials and methods

### Animals and drug exposure scheme

The experiment was approved by, and carried out in accordance with the guidelines of the Norwegian Committee for Experiments on Animals (Forsøksdyrutvalget, FDU), following standardised application through the animal facility at Haukeland University Hospital (ID 2014–6735). Ten to 12 weeks old female outbred Sprague-Dawley rats (weighing ~240 g) (Mollegaard, Denmark) were housed under standard conditions with an artificial 12:12 h light/dark cycle under constant 48% humidity. Female rats were selected as the administration of long-acting olanzapine has resulted in clinically relevant metabolic phenotypes, while exposure of male rats have yielded less clear-cut results^26–28,31^. Five animals were housed in each cage, with free access to standard laboratory chow (Special Diets Services, Witham, UK) and tap water. Animals were randomly divided into two groups (*n* = 10) (the investigators were blinded to the group allocations); the first group received a single intramuscular injection of commercially available olanzapine pamoate depot formulation (100 mg/kg BW ZypAdhera^®^) (Eli Lilly, Indianapolis, IN, USA), while the second group received commercially available vehicle solution (injection volume 160 μl/250 g BW) on the first day of the experimental period, as previously described^28^. Sample size and the injected dose were determined based on previous experiments^26,28^. Food was removed 10 h before sacrifice, in order to reduce potential variability in gene expression caused by differences in metabolic parameters. Based on previous rat experiments of gene expression in peripheral tissues, a time period of 5 days was selected, representing a sub-acute exposure time^26,28^. Care was taken to ensure minimal suffering of the animals at all stages of the experiments.

### Tissue dissection

Rats were anesthetised by isoflurane gas (Isoba vet; Schering-Plough, Denmark), and subsequently sacrificed by decapitation. Brains were removed from the skull, washed in cold phosphate-buffered saline and placed on ice. Tissue samples from the fronto-medial part of the cortex (FMCx), striatum and hippocampus were dissected from the right hemisphere. In addition, the whole hypothalamus was dissected. Tissue samples were immediately frozen on dry ice, and stored at −80 °C.

### Measurement of plasma olanzapine levels

Truncal blood was collected as previously described^26^. Plasma levels of olanzapine were determined by means of high-performance liquid chromatography using Agilent 1290 Infinity Binary LC, coupled to an Agilent 6490A triple quadrupole mass spectrometer using positive electrospray ionisation (Agilent Technologies, Santa Clara, CA, USA).

### RNA extraction

Tissue samples from FMCx, hypothalamus, hippocampus and striatum were homogenised using a TissueLyser (Qiagen, Hilden, Germany). RNA extraction was performed on an ABI Prism™ 6100 Nucleic Acid PrepStation (Applied Biosystems, Foster City, CA, USA), with DNase treatment according to the manufacturer’s protocol. Amount and quality of total RNA was measured using the NanoDrop Spectrophotometer (NanoDrop Technologies, Wilmington, DE, USA) and the Agilent 2100 Bioanalyzer (Agilent Technologies). All samples had an RNA integrity number above 7.5.

### cDNA synthesis and quantitative real-time PCR

cDNA synthesis and quantitative real-time-PCR (qPCR) were performed as previously described^26,32^. Relative gene expression levels were determined by the comparative C_T_ method (ΔΔC_T_), using *Acidic ribosomal phosphoprotein P0* (*Arbp*) and *β*-*actin* (*Actb*) as endogenous controls. The two-sided Student´s *t*-test was used to assess statistical significance between groups (*p*-value < 0.05).

### Microarray experiments

The microarray experiment was performed using the Agilent Technologies Rat Gene Expression 4x44K v3 Microarray Kit (Agilent Technologies). One hundred and fifty nanograms total RNA from each FMCx sample was reversely transcribed, amplified and Cy3-labelled using the Agilent Low Input Quick Amp Labelling Kit, one-colour v.6.6. 1.65 μg of Cy3-labelled cRNA was hybridised to Agilent Rat Gene Expression 4x44K v3 Microarrays, which were subsequently washed according to the manufacturer’s instructions. The Rat Gene Expression 4x44K v3 Microarrays contain 45,018 probes representing 26,930 rat genes, sourced from RefSeq Build 36.2, Ensembl Release 55, Unigene Build 177 and GenBank (January 2009). Fluorescent signal detection was performed by the Agilent Technologies Scanner G2505B, and the resulting images were processed by Agilent Feature Extraction Software version 10.7.

### Microarray data analysis

Signal intensities were imported into J-Express 2012 (Molmine, Bergen, Norway)^33^. To minimise the effect of external technical variables (e.g. RNA extraction, labelling and hybridisation) and to obtain a normal distribution, inter-array quantile normalisation and log2 transformation was performed, respectively. Identification of single microarray probes that differed significantly in expression level (i.e. hybridisation signal intensity) between sample groups was carried out by significance analysis of microarrays (SAM)^34^, comparing signal intensities of all probes across all samples. In order to reduce the number of false-positive findings, the significance threshold was set to a highly conservative false discovery rate (FDR) of 0^35^ and 1000 permutations were performed. Statistically significant gene expression profiles were further explored and curated by manual inspection. Gene set enrichment analysis (GSEA) was used to analyse the global gene expression data, at the gene-set level. A priori defined gene sets were based on Gene Ontology (GO) annotations, implemented in J-Express^33^. In GO, genes are classified along three domains, namely molecular function (i.e. activity of gene products), cellular component (i.e. site of active gene product) and biological process (i.e. pathways comprising the activities of multiple gene products), and each domain is further sub-divided into a hierarchical structure. In addition, each GO annotated gene has a defined relationship to other genes within the same domain, or to other domains, indicating that there is some overlap between the three domains^36,37^. In order to reduce false-positive—and negative—findings, we implemented highly stringent analysis settings: 25–200 genes per gene set, 5000 permutations and FDR ≤ 25.

### Analysis of leading edge genes

“Leading edge” (LE) genes, i.e. the genes driving the enrichment score in the GSEA method, were exported and explored through Qiagen´s Ingenuity^®^ Pathway Analysis (IPA) (Qiagen, Redwood City, CA, USA).

## Results

### High plasma concentrations of olanzapine

Five days after a single injection of long-acting olanzapine formulation (100 mg/kg), high and therapeutically relevant olanzapine plasma concentrations were determined, ranging between 66.1 and 229.1 nM in the animals (*n* = 10) (mean ± S.E.M: 119.7 ± 15.8 nM).

### Olanzapine exposure leads to few changes at the single gene level in global gene expression in rat fronto-medial cortex

**Fig. 1:**
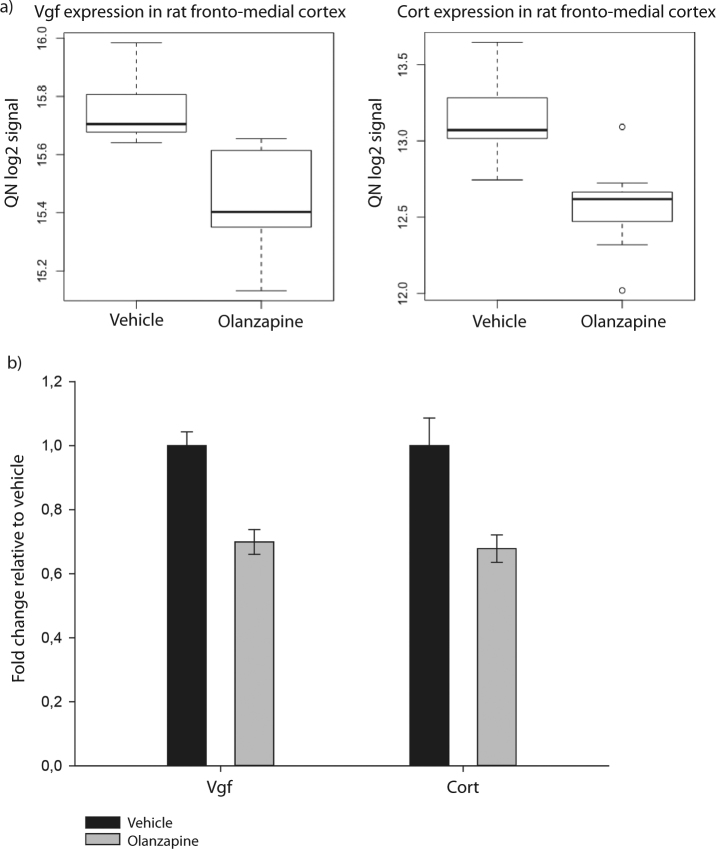
Downregulation of genes in rat fronto-medial cortex in response to olanzapine exposure **a** SAM of global gene expression in FMCx from rats exposed to olanzapine (*n* = 10), compared to control animals (*n* = 10), revealed downregulated expression for two genes, Vgf and Cort. The box plots illustrates the relative expression levels (quantile normalised, log2-transformed singal intensities) of Vgf (left) and Cort (right), where the median of the distribution (thick black line), 75th percentile (upper edge of box), 25th percentile (lower edge of box), 95th percentile (upper edge of vertical line), 5th percentile (lower edge of vertical line) and the outlier points (above and below vertical lines) are indicated. **b** qPCR analysis revealed downregulated Vgf and Cort expression in response to olanzapine exposure (fold change: −1,43 and −1,47; *p*-values: 0.0013 and 0.004 (two-tailed Student's *t*-test), respectively). The *y*-axis indicates fold change relative to control animals. Genes are placed on the *x*-axis; samples from control animals (*n* = 10) (vehicle) in black, samples from olanzapine-exposed animals in grey (*n* = 10). Data are given as mean ± S.E.M.

SAM was used to identify differentially expressed single genes in the global gene expression data in FMCx from olanzapine-exposed animals (*n* = 10), vs. controls (*n* = 10). At the global level, two genes were found to display significant downregulation (FDR = 0); the neuropeptide precursor encoding gene Vgf, and the neuropeptide-encoding gene Cort (fold change −1.25, and −1.48, respectively) (Fig. 1a; for gene expression profiles across all samples, see Supplementary Figure 1). The differential gene expression was verified by qPCR (fold change: −1,43 and −1,47, *p*-value: 0.0013 and 0.004, for Vgf and Cort, respectively) (Fig. 1b). Of note, the third lowest FDR value in the SAM analysis was 70, far from the defined significance threshold (Supplementary Table 1).

We further examined whether the two neuropeptide-encoding genes displayed a similar downregulation in three additional brain regions, namely hippocampus, striatum and hypothalamus. Using qPCR analysis, we found that Vgf, but not Cort, was downregulated in hippocampus in response to olanzapine exposure (fold change: −1,91, *p*-value: 0.017, data not shown). None of the two genes were differentially expressed in striatum or hypothalamus.

### Myelination-related genes are upregulated at the gene-set level in rat fronto-medial cortex in response to olanzapine exposure

**Fig. 2:**
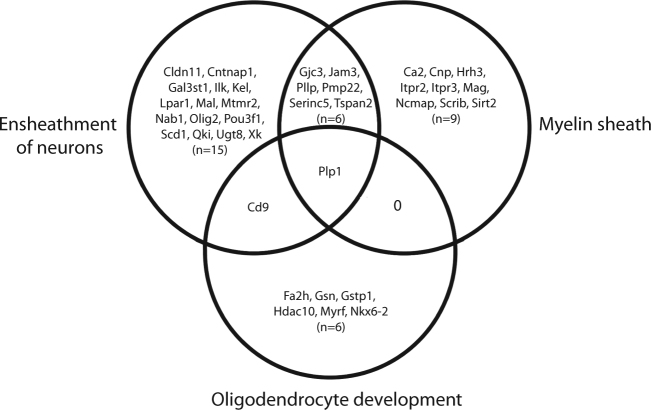
Overlap between myelination-related gene sets Venn diagram illustrating the overlap between leading edge (LE) genes from the three gene sets “Ensheathment of neurons”, “Myelin sheath” and “Oligodendrocyte development”. The gene symbol and number of overlapping genes, from each gene set, are listed in the intersection between gene sets. 0 indicates no overlapping genes. The figure was generated by Ingenuity Pathway Analysis (IPA) tool

GSEA was performed to identify potential sets of genes with altered expression in response to olanzapine exposure. This method is useful in detecting moderate cumulative changes, in genes with an a priori defined relationship (e.g. based on GO terms), which taken together can explain changes in expression that would not reach a conservative significance threshold in single gene analysis. Under highly stringent settings (see Materials and methods), a total of 1964 GO predefined gene sets were tested, resulting in six gene sets identified as significantly changed in FMCx in response to olanzapine exposure (Table 1). Five of these gene sets were upregulated in olanzapine-exposed animals, and strikingly, all of them were linked to oligodendrocytes and myelination processes; “Ensheathment of neurons” (FDR = 13,1), “Axon ensheathment” (FDR = 8,8), “Myelination” (FDR = 19,1), “Myelin sheath” (FDR = 20,3) and “Oligodendrocyte development” (FDR = 22,6) (Table 1). The genes with a positive contribution to the enrichment score (i.e. the “leading edge” (LE) genes) for each gene set (Supplementary Table 2) were further explored. The LE genes for the gene sets “Ensheathment of neurons”, “Axon ensheathment” and “Myelination” were found to be identical, and subsequent analysis was performed using the “Ensheathment of neurons” term (*n* = 23 genes) to represent the three sets of genes. The LE genes from the two remaining gene sets, “Myelin sheath” (*n* = 16 genes) and “Oligodendrocyte development” (*n* = 8 genes), did not share any similarity, except for one gene, Plp1, which was common between all three gene sets (Fig. 2). The most overlap was found between the “Ensheathment of neurons” and the “Myelin sheath” gene sets, sharing a total of six genes.

**Table 1:** Gene set enrichment analysis of fronto-medial cortex in animals exposed to olanzapine, compared to control rats

Gene set	# Genes	*p*-val	FDR	Leading edge genes
Upregulated				
Ensheathment of neurons	59	0.00	13,1	*n* = 23, Cd9, Cldn11, Cntnap1, **Gal3st1**, Gjc3, Ilk, Jam3, Kel, Lpar1, **Mal**, Mtmr2, Nab1, Olig2, Pllp, **Plp1**, Pmp22, Pou3f1, Qki, Scd1, **Serinc5**, **Tspan2**, Ugt8, Xk
Axon ensheathment	59	0.00	8,8	*n* = 23, Cd9, Cldn11, Cntnap1, **Gal3st1**, Gjc3, Ilk, Jam3, Kel, Lpar1, **Mal**, Mtmr2, Nab1, Olig2, Pllp, **Plp1**, Pmp22, Pou3f1, Qki, Scd1, **Serinc5**, **Tspan2**, Ugt8, Xk
Myelination	57	0.00	19,1	*n* = 22, Cd9, Cldn11, **Gal3st1**, Gjc3, Ilk, Jam3, Kel, Lpar1, **Mal**, Mtmr2, Nab1, Olig2, Pllp, **Plp1**, Pmp22, Pou3f1, Qki, Scd1, **Serinc5**, **Tspan2**, Ugt8, Xk
Myelin sheath	48	0.01	20,3	*n* = 16, Car2, **Cnp**, Gjc3, Hrh3, Itpr2, Itpr3, Jam3, **Mag**, Ncmap, Pllp, **Plp1**, Pmp22, Scrib, **Serinc5**, Sirt2, **Tspan2**
Oligodendrocyte development	33	0.00	22,6	*n* = 8, Cd9, **Fa2h**, Gsn, **Gstp1**, Hdac10, **Myrf**, **Nkx6-2**, **Plp1**
Downregulated				
Protein localisation in endoplasmic reticulum	26	0.00	17,3	*n* = 14, Ankrd13c, Bcap31, Kdelr1, Kdelr3, Macf1, Os9, Ryr2, Sec16b, Sec61a1, Srp19, Srp54a, Srp9, Srpr, Ubac2

Significance threshold was set to FDR < 25. Gene sets were derived from Gene Ontology (GO), organised with “ancestor” gene sets listed first, followed by “children” when present. *p*-val: nominal *p*-value, where a *p*-val = 0.00 indicates a *p*-value less than 1/5000 (one divided by the number of permutations performed). *FDR*, false discovery rate. Number of LE gene members in each gene set is listed in the Leading edge genes column. Genes highlighted in bold indicates the top five ranked genes from each gene set, in addition to the overlapping genes, analysed by qPCR.

**Fig. 3:**
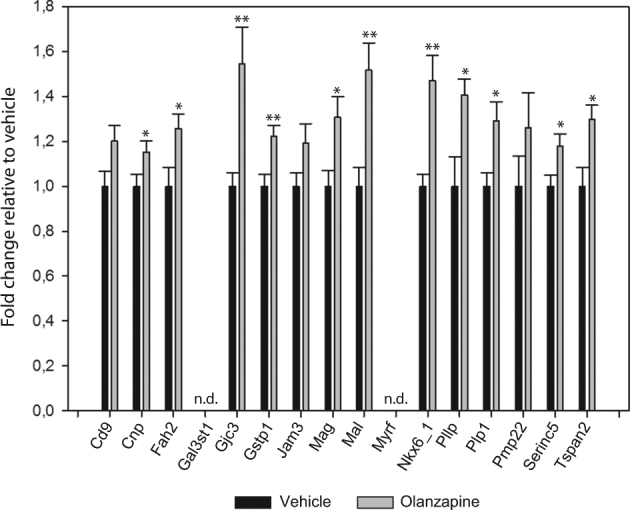
qPCR analysis of “Leading Edge” genes Sixteen LE genes, selected based on their rank in the gene set, and overlap between gene sets, were analysed by qPCR. Eleven out of the selected LE genes were significantly upregulated in response to olanzapine exposure in the FMCx. Gene symbols are listed at the *x*-axis, while the *y*-axis indicates fold change relative to control animals. Samples from control animals (*n* = 10) (vehicle) in black, samples from olanzapine-exposed animals (*n* = 10) in grey. Data are given as mean ± S.E.M. *: *p*-val < 0.05, **: *p*-val < 0.01, n.d.: not detected, as determined using two-tailed Student's *t*-test

Although none of the LE genes from the myelination-related gene sets identified through the GSEA method were found to be significantly different in the single gene-based SAM analysis (see above) many of them were indeed upregulated upon independent re-examination. We analysed a subset of these genes by qPCR, i.e. the top five LE genes from each gene set, in addition to the LE genes showing overlap between the three gene sets, resulting in a total of 16 unique genes (highlighted in bold in Table 1 and listed in Supplementary Table 3). Of these, we were able to show significant upregulation of 11 genes (Fig. 3), namely Plp1, Serinc5, Tspan2, Fa2h, Mag, Mal, Gjc3, Gstp1, Nkx6.2, Pllp and Cnp. Two other genes, Cd9 and Jam, displayed a trend towards upregulation (*p*-values: 0,057 and 0,093, respectively). The remaining three genes were either not detected (i.e. Gal3st1 and Myrf), or did not reach the significance threshold (i.e. Pmp22).

In the GSEA, only one gene set was found to be downregulated in the FMCx, and this gene set was linked to translational processes (“Protein localisation in endoplasmic reticulum”, Table 1).

### Effect of olanzapine exposure on myelination-related genes in hippocampus, striatum and hypothalamus

Five of the LE genes ranked as most significant in the three genes sets, namely Serinc5, Tspan2, Cnp, Fa2h and Plp1, were further analysed by qPCR in samples from hippocampus, striatum and hypothalamus. Only one gene, Serinc5, was found to display a similar upregulation in the hippocampus as in FMCx (fold change: 1,23, *p*-val: 0,023). No differential gene expression was observed in the hippocampus for the four other genes in response to olanzapine exposure. In striatum and hypothalamus, none of the selected LE genes displayed differential gene expression patterns in response to olanzapine exposure (data not shown).

## Discussion

In this study we analysed alterations in global gene expression in the rat fronto-medial cortex, induced by olanzapine at stable and clinically relevant serum concentrations. A marked upregulation of genes linked to oligodendrocytes and myelination processes was identified, in addition to changes in two neuropeptide-encoding genes.

Of the ~2000 GO predefined gene sets analysed by GSEA, we observed an upregulation of five gene sets, all of which were linked to oligodendrocyte development and myelination. Three of the gene sets shared the same LE genes, and were thus treated as similar, resulting in a total of three different myelination-related gene sets, namely “Ensheathment of neurons”, “Myelin sheath” and “Oligodendrocyte development”. Both “Ensheathment of neurons” and “Oligodendrocyte development” belong to the GO defined biological process domain, while “Myelin sheath” is classified as belonging to the cellular component domain. Myelination has received increasing attention in schizophrenia research over the last decades. Post-mortem-, but also imaging- and genetic studies, have provided evidence for abnormalities in white matter and myelin^38–45^. One of the most consistent findings from global gene expression studies in post-mortem brain samples from patients suffering from schizophrenia has been downregulation of genes related to normal oligodendrocyte function and myelination^12–16^. Hakak et al.^13^ were the first to report reduced expression of myelination-related genes in the dorsolateral prefrontal cortex from elderly schizophrenic patients. Interestingly, out of the six genes reported to be downregulated by Hakak et al., four were among the upregulated LE genes identified in our analysis (i.e. Mag, Mal, Cnp and Gsn). In a different study, three genes were found to be downregulated in the temporal cortex of patients suffering from schizophrenia, and two of these were among the LE genes identified by us, namely Mal and Pllp^12^.

Interestingly, a recent study found that oligodendrocyte-related gene sets (based on literature studies and GO annotations, i.e. “lipid metabolism”, but also “oxidation-reduction”, and “gene transcription”) were significantly associated to the risk of developing schizophrenia^46^. Most of the genes comprising the “Lipid metabolism” gene set were shown to be involved in metabolism of structural membrane lipids of the myelin sheath, with additional roles in sorting, trafficking and anchoring of myelin proteins in the myelin membrane^46^.

In our analysis, two of the upregulated gene sets, “Ensheathment of neurons” and “Myelin sheath”, comprised LE genes primarily linked to structural components of the myelin sheath. Serinc5 was ranked as the most enriched gene in both of these two gene sets. The encoded Serinc5 protein is an important factor in facilitating and regulating biosynthesis of myelin glycolipids, which is a major component of myelin (27% of the total lipid content)^47^. The encoded Serinc5 protein localises to endoplasmatic reticulum membranes, where it binds directly to lipid biosynthetic enzymes also embedded there, incorporating the nonessential amino acid serine into glycolipids synthesised in oligodendrocytes^47^. In addition, the “Ensheathment of neurons” gene set, and to a lesser extent the “Myelin sheath” gene set, comprised several genes encoding tetraspans (membrane-embedded proteins with four transmembrane domains), i.e. Tspan2, Mal, Pllp, Cd9, Pmp22 and Plp1. Such tetraspans constitute a large fraction of myelin proteins, and have a role in formation of membrane junctions and regulation of growth and migration of myelin-producing cells^48^. Plp1, the only LE gene found in all three gene sets, has previously been shown to display almost exclusive expression in newly formed- and mature myelinating oligodendrocytes, constituting the most abundant myelin tetraspan protein in the central nervous system^48–50^. The encoded protein has an important role in the biogenesis and structure of myelin and maintenance of myelin sheaths, in addition to oligodendrocyte development and axonal survival^51^. The third gene set found to be upregulated in response to olanzapine in the FMCx, “Oligodendrocyte development”, comprised mainly LE genes encoding intracellular proteins, such as the transcription factor Nkx6.2. This factor has previously been implicated in regulation of the myelination process, being expressed in differentiating, but also in newly formed and mature oligodendrocytes^50,52^. It has multiple binding sites in the promoter regions of Mbp and Plp1, further supporting that this factor could be involved in regulating myelin-specific gene expression^52,53^.

The transcriptional upregulation of genes linked to oligodendrocyte development and structural components of myelin in response to olanzapine exposure could suggest that one of olanzapine’s antipsychotic actions is to compensate for abnormal myelination, e.g. by activating dormant oligodendrocyte progenitor cells into mature myelin-producing oligodendrocytes and promote myelin integrity^54^. We did not observe such an upregulation outside cortical areas in our studies, suggesting that this effect may be restricted to the cortex. In fact, only one gene, Serinc5, showed increased expression elsewhere, namely within the hippocampus.

Indeed, imaging studies have demonstrated increased white matter volume in the cerebral cortex in response to antipsychotic medication, primarily due to increased myelination at deeper cortical levels^55,56^. Studies in animals receiving antipsychotic medications have also demonstrated morphological changes in the brain, including increased glial density in deeper cortical layers^57^. Furthermore, gene expression studies in mice have reported on alterations in lipid-related genes in response to the antipsychotics clozapine and haloperidol in cortex, but also to a certain extent in striatum^58^. Finally, cuprizone-induced demyelination in mice can be prevented by exposure to the second-generation antipsychotic quetiapine, which also promoted oligodendrocyte development^59^. Although most work in the field has been focusing on quetiapine, one study also examined the effect of olanzapine in cuprizone-induced demyelination in mice. Here, olanzapine was found to effectively decrease myelin breakdown and loss of oligodendrocytes, in response to cuprizone exposure in mice^60^. Further studies should aim at resolving whether the olanzapine induced upregulated expression of genes linked to oligodendrocytes and myelination processes is a general effect, by comparing different antipsychotic drugs head to head. In addition, such studies should also determine whether the effect could be observed at several different time-points. Our study did not consider the potential confounding effect of the oestrous cycle stage, which has previously been demonstrated to influence gene expression in rat medial prefrontal cortex^61^. However, none of the individual genes found to be differentially regulated in our study seemed to be affected by oestrous stage^61^.

At the single gene level, we found that the two neuropeptide-encoding genes Vgf and Cort were significantly downregulated in the rat FMCx in response to olanzapine exposure. In the brain, Vgf is widely distributed, but shows particular abundance in distinct areas, such as the cerebral cortex, hippocampus, olfactory system and hypothalamus^62,63^. The encoded protein gives rise to several low molecular weight neuropeptides, and has been implicated in regulation of energy balance^64,65^. However, the exact function of the majority of Vgf neuropeptides remains elusive. In our study, expression of Vgf was also downregulated in hippocampus, but no alterations were observed in the striatum or hypothalamus. The second identified gene, Cort, is primarily expressed by GABAergic interneurons, and has a scattered expression pattern across the cerebral cortex^66^. In our study, this gene was downregulated in FMCx after olanzapine exposure. Stable expression levels within hippocampus, striatum and hypothalamus could indicate a region-specific drug effect for this gene as well, as previously discussed for the myelin-related gene sets. The encoded neuropeptide has been linked to learning and memory processes and cortical synchronisation, but has also been suggested as a mediator of immunity and inflammation^67^. The link to immunity and inflammation is especially interesting, since such processes have been implicated in the pathophysiology of schizophrenia^6,68,69^. It is possible that olanzapine may in part lower inflammatory responses, by reducing the expression level of Cort.

In summary, we have demonstrated that exposure to long-acting olanzapine leads to significant upregulation of myelination-related gene sets and downregulation of two neuropeptide-encoding genes in rat FMCx. To the best of our knowledge, this is the first large-scale gene expression study identifying alterations in genes linked to myelination-related pathways, in response to antipsychotic exposure in rats. Several of the upregulated myelination-related genes identified by us have previously been reported as downregulated in post-mortem brain samples from patients diagnosed with schizophrenia. Antipsychotic-induced induction of myelination-related gene expression may be relevant both to the clinical effects of antipsychotic agents and to the pathophysiology of schizophrenia.

## Supplementary information

Gene expression profiles
Top hundred SAM results
“Leading Edge” genes from myelination- and oligodendrocyte- related gene sets identified by GSEA
“Leading Edge” genes for qPCR validation
