# Supplementary material for: Subchronic olanzapine exposure leads to increased expression of myelination-related genes in rat fronto-medial cortex
Source: Transl Psychiatry. 2017 Nov 30;7:1262. doi: 10.1038/s41398-017-0008-3 (PMC5802494; doi:10.1038/s41398-017-0008-3)
Supplement: Supplementary file 1 — Gene expression profiles [file 41398_2017_8_MOESM1_ESM.docx]

**SUPPLEMENTARY MATERIAL**


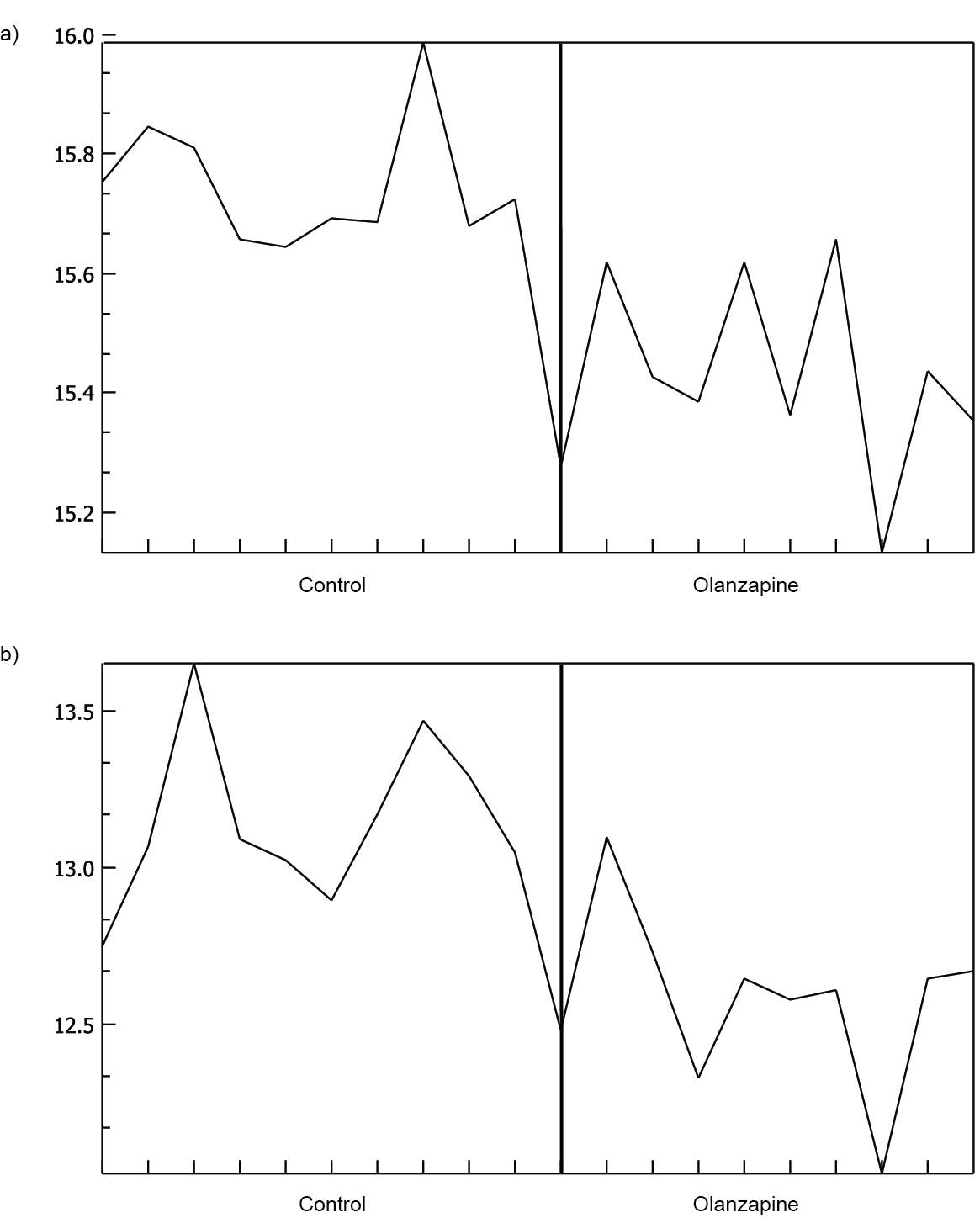


**Supplementary Figure 1**: **Gene expression profiles** **a)** *VGF nerve growth factor*, and **b)** *Cortistatin,* found to be differentially expressed in FMCx from olanzapine exposed rats. Individual samples are placed on the x-axis; control samples to the left, olanzapine samples to the right, border is indicated by a solid line. The y-axis indicates normalised signal intensities for the gene in each individual sample. Profiles were generated in J-Express.
